# Historical perspectives on forestry science and monocultures: Ideas of rationality in Sweden during the early twentieth century

**DOI:** 10.1007/s13280-024-01987-9

**Published:** 2024-02-28

**Authors:** Jimmy Jönsson

**Affiliations:** Department of Arts and Cultural Sciences, Lund University, Box 192, 221 00 Lund, Sweden

**Keywords:** Environmental history, Forestry, History of ideas, Mixed forests, Monocultures, Sustainable yields

## Abstract

This study aims to broaden our historical knowledge about ideas of rationalism and monocultures in forestry science and rational forest management. Empirically, it focuses on the writings of Swedish forestry scientist Henrik Hesselman, active in the early twentieth century. The texts were analyzed using the method of historical contextualization. The study indicates that monocultures historically have been subjected to debates richer than what previous research gives credit for. Besides a rationalist technology, monocultures have been conceptualized as an example of non-rational forestry failing to deliver sustainable yields. Moreover, instead of only simplifications, one-size-fits-all solutions, and top-down reforms, historical forestry science representatives have also at times understood rational forest management as a quest for complexity, site-specific solutions, and bottom-up approaches. It is argued that our understanding of forest use and society–environment relations, more generally, benefit from more historical contextualization.

## Introduction

Most of the world’s cultivated lands—be they fields, orchards, or forests—are monocultures, that is, stands of one single species. Yet, despite their distribution, the technology of monocultures is controversial (e.g., Balough 2021). While its advocates stress advantages in profit and planning, its opponents point toward diseases, impoverished soils, and threats to biological diversity.

This is a historical study analyzing ideas of monocultures in the context of forestry science and rational forest management. Monocultures have been a vital part of foresters’ repertoire since the emergence of rational forest management in the eighteenth century (Lowood 1990; Scott 1998; Puettmann et al. 2008). Mimicking the farmer’s field, containing only one crop species, foresters have kept growing stands comprising one tree species for benefits in yields and planning. However, although not as polarizing as today, monocultural forests have been subjected to debates for a long time; much longer than key concepts like “biological diversity” or, for that matter, “monoculture” has been around. For example, already in the 1880s, foresters raised warnings for diseases and soil impoverishment following the planting of monocultures in German forests (Hölzl 2010).

The history of monocultures in forestry science and rational forest management has mainly been told through studies of “reductionist foresters” (e.g., Scott 1998; Puettmann et al. 2008). By reductionist forestry, I refer to foresters associating the idea of rationality and goal of maximizing forest yields with a set of idiomatic top-down reforms, one-size-fits-all solutions, and technologies minimizing diversity and promoting uniformity and simplification. In practice, this has meant an indivisible “management package” of means like clearcutting, even-aged stands, and, not least, monocultures. Promoting this, reductionist foresters are argued to have aimed for uniform forests efficiently reducing away any non-profitable “weeds,” meticulously regulated using the maps and tablets at their offices. Most influentially, reductionist forestry has been studied as a “high modernist” ideology by James C. Scott (1998).

Historically, forestry science and rational forest management have indeed had its fair share of simplifications, one-size-fits-all solutions, and top-down reforms (Demeritt 2001; Radkau 2012). However, to solely connect the historical rationalism of rational forest management with monocultures and other reductionist technologies and group a disparate crowd of actors—from different periods, of different nationalities, engaged in different political projects—is problematic. The approach might give an accurate image of the spokesmen of monocultures but threatens to limit our knowledge about the technology’s actual historical status. As others have shown, our understanding of past forest use and society–environment relations more generally could benefit from more empirical nuances and historical contextualization (e.g., Langston 1995; Hölzl 2010; Loo and Stanley 2011; Mårald et al. 2016; Jönsson 2019).

This study aims to broaden our historical knowledge about ideas of rationalism and monocultures in forestry science and rational forest management. As an empirical case, I use a historical individual’s ideas of rationality, monocultures, and mixed forests, that is, the ideas of the Swedish forestry scientist Henrik Hesselman (1874–1943). Following international trends of increased collaborations between natural science and natural resource management during the early twentieth century, Hesselman was dedicated to the building of an extensive Swedish forestry sector aiming for the maximization of forest yields (Jönsson 2019). He operated from a governmental forestry science institution, the Swedish Institute of Experimental Forestry (created in 1902) as a researcher and, in time, director. Here, he elaborated research later used to support large-scale applications of fertilizers and clearcutting in Swedish forests. He also contributed to the creation of a forest inventory institution, mapping the economic capacity of Sweden’s forests. Additionally, he had bonds to the forest industry and invested a great deal of energy into forming public opinion for industrial use of Sweden’s forests. Hesselman was however not a reductionist. Focusing on how he associated rationality with monocultures and mixed forests, the study will demonstrate that reductionist features such as simplification, one-size-fits-all solution, and top-down reforms clashed with his mindset.

The study also demonstrates the utility of history. Monoculture policy ought to be founded on multidisciplinary knowledge from natural, social, and humanistic sciences. Having a good track record of being policy relevant (Sörlin 2011; Hughes 2016), environmental history could be expected to partake in the monoculture debate. Yet, with few exceptions (e.g., Uekötter 2011), historians have been absent in this context.

After the Materials and Methods section below, I sketch a background about the Swedish Institute of Experimental Forestry and Hesselman’s work. Thereafter, I demonstrate my analysis of Hesselman’s writings and discuss, subsequently, different contextual interpretations of the texts. Finally, the Discussion and Conclusion section follows.

## Materials and methods

The analysis was based on readings of all articles, speeches, pamphlets, and other texts from Hesselman meeting the study’s aim (Hesselman 1904, 1905, 1906, 1917a, b, c, d, 1919, 1922, 1925, 1937). The corpus stretches from scientific papers to popular science outlets. The latter publications were intended to persuade foresters, industrialists, forest owners, and others on management methods or ways of understanding the forest. All texts are available at the National Library of Sweden.

The reading process was divided into two steps. First, I interpreted the texts using their historical context as explanation. This method demands that the researcher consider the historical author’s intentions in relation to contemporary political priorities and other factors in the historical context (Skinner 2002). In this case, such factors were constituted by debates, political currents, and Hesselman’s biography and network. The context was reconstructed from previous research but also from historical sources. Second, having established a contextual interpretation, I looked for overarching trends. The rationale behind this step was to separate temporary whims from robust themes in Hesselman’s thinking.

## Results

### Launching of experimental forestry science in Sweden

Hesselman’s career took place against the backdrop of intense debates about forest regeneration. Since the late nineteenth century, Swedish foresters, politicians, state administrators, and others had debated obstacles to the securing of forestry as a main national industry by means of sustainable-yield forestry (Mårald and Westholm 2016; Mårald et al. 2017; Eliasson and Törnlund 2018). This meant, for several debaters, that both private- and state-owned forests should be used as eternal industrial assets where the forest’s cutting rate would never exceed its re-growth rate. Still, the national wood supply seemed to diminish; the forests did not regenerate as they should have. Many put the blame on a timber frontier going north during the nineteenth century (Josefsson and Östlund 2011). Sawmills, pulp industries, and other commercial actors seemed to have cut far too large areas without caring for re-growth measures. The application of sustainable-yield forestry was also prevented by other older types of forest use, such as farmers’ and indigenous Sámi populations’ use of forests for grazing and wood supplies (Östlund and Norstedt 2022). Furthermore, no consensus on sustainable forestry methods existed which led to heated debates about ditching, clearcutting, and tree planting (Eliasson 2010; Lisberg Jensen 2011). The regeneration debate resulted in several political actions, most noteworthily, the forestry acts of 1903 and 1923 steering forest owners toward sustainable-yield forestry (Appelstrand 2007).

Also, following a historical patterns, the regeneration debate implied that foresters, politicians, industrialists, and others should look for *scientific* answers about forest management (Eriksson 1978; Bruno and Lundin 2020). The late 19th and early twentieth century in Sweden therefore saw the creation of a professor’s chair in forest biology (in 1897), the Swedish Institute of Experimental Forestry (1902), the Royal College of Forestry (1914), experimental forestry research stations (1921–1923), and the Swedish National Forest Inventory (1923). The Swedish Institute of Experimental Forestry aimed at researching issues preventing “rational forest management” (Jönsson 2019). This included, for example, investigating hardiness of tree “races,” national conditions for tree planting, and Swedish “forest types,” as well as conducting basic research about “natural laws of forestry.” Here, Hesselman exemplified international trends where biologists and ecologists started to engage in efficient natural resource management (Worster 1994).

Hesselman spent his entire career at the Institute, though developed international networks primarily with German and Danish foresters (Söderqvist 1986; Jönsson 2019). He came to the institution as a young man in 1902 and worked on a doctoral thesis about wood meadows based on studies in an archipelago north of Stockholm. He was trained as a laboratory botanist but had strong interests in field science. The latter interest impelled him to transport lab equipment out into the forest as much as to bring samples back to the laboratory. After a few years, he became the Institute’s chief botanist and, in the 1920s, the Institute’s director, a post at which he remained until his retirement in the late 1930s. Many of the Institute’s research tasks were initially performed by Hesselman himself, which is why he developed a repertoire stretching from genetics, ecology, and microbiology to mathematical statistics. As the Institute grew, he hired assistants to specialize in different fields. This allowed him to focus on humus research and on searching for answers about forest regeneration in the forest ground’s top layer—a field where he received international recognition. Throughout his career, Hesselman also participated in the works of the Swedish Forestry Society. This organization tried to form opinion in favor of industrial interests and sustainable-yield forestry in opposition to agrarian forest use, such as forest grazing. It was led by scientists, industrialists, large landowners, and politicians and operated mainly through journals and other printed outlets.

The words “rational” and “rationalization” were novel buzzwords in Sweden at the time (although they have a longer history as technical terms in mathematics and philosophy). They were introduced in the late 1910s to efficient factory work, but soon found their way into several other societal sectors replacing words like “ordered” and “scientific” (Björck 2008), much like everything is labeled “smart” today. As shown below, Hesselman used the word rational frequently. From the 1920s, he likely connected his task to the contemporary technocratic discourse intentionally. Back at the turn of the century, however, he probably used rational less consciously and could as easily have talked about “ordered” or “scientific.”

### Hesselman’s ideas of rationality, monocultures, and mixed forests

Hesselman returned to questions of rationality, monocultures, and mixed forests throughout his career. The first stop of the analysis is a set of texts published in the twentieth century’s first decade (Hesselman 1904, 1905, 1906). Back then, Hesselman had just acquired a doctoral degree and begun his employment at the Swedish Institute of Experimental Forestry.

One of the first things he wrote as a forestry scientist was a popular science paper about wood meadows for a Swedish Forestry Society journal (Hesselman 1905). Here, he stressed that his studies were “ecological” in the sense of focusing on “organisms’ dependence on the outer life conditions,” such as “the soil’s constitution, access to light, and air humidity” (p. 13, 14). He emphasized that wood meadows are constituted by a high number of tree species and other plant species living in integrated relationships with each other. Hesselman saw his ecological approach to wood meadows as beneficial to the Swedish forestry sector. To reduce Sweden’s dependence on imported wood from species, such as ash, oak, and hornbeam, he argued that the nation ought to submit its wood meadows to a “fully rational” treatment (p. 22–23). The Swedish forestry sector should thus commence cultivating mixed forests of different compositions. He exemplified with one type of stand providing the industry with “ash, oak, maple, alder, and elm” and another with “ash, German maple, alder, oak, elm, beech, hornbeam, field maple, willow, and rowan.” Interestingly, the concept of “rational” did not designate reductionist top-down methods streamlining the forest, but *bottom-up* approaches using diversity as a point of departure:

The management [of wood meadows] must be founded on careful considerations in relation to the different tree species’ demands for light and nutrients in the soil. A mixture of different tree species should be favorable and, in many ways, a pure necessity considering the often strongly changeable constitution of wood meadow soil and the deciduous trees’ […] life needs.

These ideas—of using complexity rather than simplification, of coming from below instead of above—to form management strategies reoccurred in other contemporary texts by Hesselman. For instance, in 1906, the Swedish Forestry Society published a pamphlet by him introducing Sweden’s “forest plant communities.” Here, Hesselman stressed ecological variables as important for “ordered forest management:” “A forest consists of […] a large amount of plant species, which form a community of their own, where the different members […] are concatenated of mutual demands for heath, humidity, and soil nutrient, but which also […] are dependent of one another” (p. 8–9, 10).

A decade later, at the end of the 1910s and beginning of 1920s, Hesselman published a series of texts about “rational soil management” (Hesselman 1917a, b, c, d, 1919, 1922, 1925). He was now a professor and prominent figure in Swedish forestry science. Delegating several research tasks to assistants, he focused on investigating forest humus to find biological factors causing low and high forest regeneration. The concept of rational soil management drew on his humus research and was meant to direct the forestry sector’s attention to soil constitution. The selection of “forestry form,” Hesselman (1917a, p. 1) argued, ought to be based on its ability to “maintain or in best case increase the production capacity of the forest soil,” just like the “farmer plows, ditches, and manures” to protect and increase fertility.

As with wood meadow cultures, the point of departure for rational soil management was ecological knowledge and the forest’s biological complexity. For example, in a popular science pamphlet published by the Swedish Forestry Society, Hesselman (1917a, p. 2) emphasized that “modern soil science” sees “the soil” as “a nature’s living workshop with labor and life” crowded with “lower organisms, worms, insects, microscopical animals, fungi, and bacteria” sensitive to changing ecological factors, such as “the ground’s evaporation, the lighting, and the vegetation.”

Also as for wood meadow cultures, Hesselman framed rational soil management as a bottom-up enterprise rather than a reductionist top-down approach. His view was most vividly expressed in a set of texts describing impressions from research travels through the European continent in 1921 (Hesselman 1922, 1925). Here, he depicted a shift where “biological forest management,” taking empirical observations of the forest soil as a starting point, had begun outcompeting the reductionist and top-down forestry approach of “mathematical forest management.” The latter approach, Hesselman (1922, p. 139, 144) stated in a popular science pamphlet, focused on nothing but digits and yields and produced ordered and uniform but lifeless and low-profit monocultures of “trees standing like soldiers in straight lines.” Biological forest management was based on more holist and organicist views, and used the forest’s ecology and multifaceted life to awaken the “slumbering forces of the soil.”

Applied to German forests, biological forest management led, Hesselman (1922, 1925) stated, to the principle of *Dauerwald*, an eternal forest canopy that would never be open. German biological foresters thus promoted methods, such as selection cutting, natural regeneration, and the using of twigs as “manure.” However, according to Hesselman, the most important aspect of this approach was the use of tailor-made solutions for certain sites’ soil constitution. The conclusion led Hesselman to recommend clearcutting in the boreal parts of Sweden but not anywhere else. The soil here, he believed, was different from continental deciduous forests.’ It required an opened canopy so as the sunrays would “activate” soil bacteria to release nitrogen.

Rational soil management also spurred Hesselman to criticize monocultures as weakening the soil and to advocate mixed forests to improve fertility. Drawing on his continental observations, Hesselman (1922, p. 143) stated that “mixed forests” of “beech” and “conifers” promoted “humus layers” of “favorable constitution,” while “pure conifer stands” required powerful measurements to attain the same levels of fertility. The idea was to use beech leaves as fertilizer: “the involvement of deciduous trees in conifer forests […] facilitates the nitrification and increases the effect of infection with soils that forms saltpeter” (Hesselman 1925, p. 330). He also discussed Swedish forest stands considering these conclusions. Especially, Hesselman (1922, p. 145) was provoked by “pure fir cultures” on old beech lands in Southern Sweden—perhaps planted by farmers to apply sustainable-yield forestry—condemning them as “abominations”.

Hesselman (1937) returned to the potential fertilizing effects of species diversity later, just before his retirement in the late 1930s. Having conducted field trials analyzing fertilizing effects of nitrogen in “overaged” fir forests at a research station outside of Umeå in northern Sweden, he noted that birch leaves improved the humus layer’s nitrification processes in some coniferous tree stands. The question was not thoroughly addressed then, since he (1973 p. 529) “hoped to publish” novel takes on “the involvement of deciduous trees” separately. These envisioned publications, however, do not seem to have seen the light of day.

### Contexts of Hesselman’s bottom-up rationality

From a present-day perspective, Hesselman’s view on monocultures can be seen as an early version of forest management approaches, such as “nature-based forestry,” harmonizing goals of environmental protection and sustainable-yield forestry (e.g., Nature-based solutions … 2022). However, on closer examination, Hesselman’s views are different from what came later. Strictly developed within a rationalist vision of the forest, Hesselman launched, as shown below, his ideas about wood meadow cultures and rational soil management with the intention to make Swedish forestry more efficient and increase its yields. Bottom-up approaches were, for him, the rationalist way, while top-down approaches were the non-rationalist alternative. This conclusion is drawn from relating Hesselman’s writings to two historical contexts: currents of nature preservation and biological forest management.

To write off the potential interpretation that Hesselman aimed for preservation rather than yields, we turn to the early nature protection movement. Like in, for example, the US, Swedish nature protection was divided into “conservation,” aiming for non-exploitative use of natural resources, and “preservation,” seeking to safeguard nature from human imprints. Conservation in this sense was mainly covered by the sustainable-yield movement; at focus here is thus the preservation side of nature protection. In Sweden, the current of thought was primarily represented by the Swedish Society for Nature Conservation (formed in 1909). Preservation of those days was different from the environmental movements of the 1960s and 1970s (Larsson Heidenblad 2021). First, the early preservation movement in Sweden was an urban and bourgeois faction led by academics operating from Stockholm or Uppsala (Haraldsson 1987; Lundgren 2009). It had no grassroots engagement. Second, the preservation movement did not aim to prevent industrial expansion, as environmentalists of latter times would, but, in a non-confrontative manner, instead aimed to mitigate its most exploitative forms (Mårald and Nordlund 2020). The preservationists were often preoccupied with remote objectives of little economic interest. Third, the early preservationist movement primarily aimed to protect untouched lands and not managed forests or other areas affected by humans (Haraldsson 1987). In practice, the movement, again like US counterparts, strived to create nature reserves and national parks protecting indigenous lands functioning as a scientific reference or national “archives” for future Swedes interested in “true” Swedish nature (Sundin 2006; Lundgren 2009).

Hesselman was engaged in the preservation movement as a board member of the Swedish Society for Nature Conservation (Haraldsson 1987). During certain periods, he even served as the Association’s president and vice president. His preservationism was typical, meaning that he called for the protection of lands to safeguard both scientific and national values (e.g., Andersson and Hesselman 1907; Hesselman 1911). However, he never tried to prevent any significant industrial enterprise, as some of his contemporaries would (Lundgren 2009; Jönsson et al. 2021).

Perhaps, Hesselman’s preservationism was even trumped by his forestry engagement. For instance, during the 1918th annual meeting of the Swedish Society for Nature Conservation, Hesselman (1918) addressed the Swedish heather moorlands, a landscape subjected to tree planting for forest industrial purposes. He emphasized the dying beauty of the moorlands in a romantic manner typical of those days. The moorland gave a “peculiar” impression of “enchanting solitude,” however, only for a short time still: “the colorful image of a flowering heather moorland is not to be found anymore” (p. 78–79). This led him to suggest that a piece of moorland should be protected as a national park. However, at the same time, he enthusiastically stressed “the outcome possibilities” following tree plantations: “the more extensive such a transition of the heather moorland into forests, the more fortunate it will be from a general national economical point of view.”

Hesselman’s approach to monocultures was thus probably not an expression of preservationism. What it seems to express, however, was a commitment to the biological forest management movement. Biological forest management emerged in the German-speaking world around the 1870s and 1880s (Lowood 1990; Hölzl 2010; Grewe and Hölzl 2018). It was formed as an internal critique of what was perceived as a dominating orientation in the forestry sector. The latter was developed in the eighteenth century administration of Prussia and other German states and relied heavily, according to the biological foresters, on clearcutting and monocultures, but also on the disciplines of mathematics and statistics. The biological foresters feared that the stressing of calculations instead of biological measurements, and one-size-fits-all methods instead of site-specific solutions, had led to a far too reductionist forestry sector, threatening the forests’ fertility and thus tomorrow’s yields—a standpoint that was often merged with chauvinistic and nationalistic agendas (Imort 2005). The biological foresters therefore criticized technologies like clearcutting, monocultures, and, more generally, one-size-fits-all means as well as the custom of treating mathematics and statistics as the prime forestry science disciplines. Additionally, they emphasized the calculable aspects of forests—the stand’s age, number of trees, size of yields, et cetera—as merely one aspect of the overall task. The forester also needed, they argued, to observe the biological world and consider the interplays between, for instance, soil moisture, species composition, and soil minerals. These views implied site-specific biological research but also a holistic, and sometimes organicist, understanding of forests. Furthermore, they suggested that rational forest management, truly aiming for high yields in the long run, should be done from below, as a bottom-up inquiry. Researchers sometime see biological forest management as a successor to the contemporary movement of “close-to-nature forestry” (resembling nature-based forestry) (e.g., Bauhus et al. 2013). However, focusing on Hesselman’s context, I do not consider latter times’ development.

In Hesselman’s writings, it is easy to find phrases suggesting that he saw himself as part of the biological forest management movement. For example, in a scientific paper presenting soil biological conclusions, he (1917b, p. 927) stated that the forestry sector too long had had a “tendency” “to use statistics to solve purely biological problems” (see also above). Sometimes he also used organicist metaphors to describe the forests’ constitution. For instance, arguing that the forester ought to consider the full complexity of biological interplays when managing a forest, Hesselman (1922, p. 144) stated that the trees and soil “formed an organic wholeness.” He and his associate, forest researcher Henrik Petterson, also visited the biological forest management celebrity Alfred Möller in the German estate of Bärenthoren in 1921. Both Hesselman and Petterson wrote enthusiastically about Möller’s methods including selection cutting, mixed forests, natural regeneration, and a general striving to keep the humus’ microbiological life satisfied (Hesselman 1922; Metoder för naturlig … 1924). An impetus from the biological forest management movement thus seems to have led Hesselman to a rationality based on holistic approaches and bottom-up solutions.

In this matter, Hesselman seems to have worked in tune with his peers, also in Sweden. For instance, Petterson was a forest statistician who, with Hesselman, constructed the Swedish National Forest Inventory in the 1910s and 1920s. The Inventory cleared the way for inserting all of Sweden’s forests into an industrial cropping system. Moreover, geographer Gunnar Andersson (1903, p. 232), Hesselman’s mentor and a major advocate of transforming Sweden’s nature into goods and yields, dismissed “the old Prussian forest cultures, with their uniform stands of only one single species in straight lines of the same age.” Andersson argued that foresters ought to “meet nature,” instead of managing from above, and let the trees “themselves, through internal struggle, decide” which tree species would grow and where. Among other things, he had taken impressions from French forests where “the pine stands” constantly were mixed with “maple, lark, and fir.”

## Discussion and conclusion

This study has indicated that monocultures in forestry have been subject to debates richer than what previous research gives credit for. For example, in their study of the emergence of silviculture, Klaus J. Puettmann, K. David Coates, and Christian Messier (2008, p. 18)—though also addressing biological forest management—state that “ecological benefits and values of multispecies stands have only recently become of interest.” It is true that monocultures have functioned as a popular rationalist method in the past. However, rationalists have also, as my study shows, conceptualized them as a prime example of non-rational forestry failing to deliver sustainable yields, a standpoint they drew directly from ecological knowledge. Note that rational was a novel buzzword and therefore open to interpretation. Still, from the very beginning, it had strong associations to industrial efficiency.

This study thus concludes, in consonance with previous research (e.g., Hölzl 2010; Mårald et al. 2016), that historical forestry science representatives have also understood rational forest management as a quest oriented toward complexity, site-specific solutions, and bottom-up approaches. As such, this version of rationality opposed simplification, one-size-fits-all solutions, and top-down reforms. Studies of past forest use as well as historical society–environment relations more generally would, I argue, benefit from seeking such empirical nuances and using contextual methods. Accordingly, I also suggest that policymakers are benefited from acknowledging historical complexity, not just in nature, but in the cultural worlds of science.

Future research into the history of monocultural forests would benefit from comparisons in time and space exploring relations between theory and practice. For example, during the twentieth century, the Swedish forestry sector developed into strongly favoring monocultures. This is not related to Hesselman’s lack of influence, but dramatical technological differences before and after World War Two. Hesselman addressed mixed forest to solve regeneration issues in a world of axes, horses, and handsaws. The postwar world saw the rise of mechanized forestry and thus other means to increase fertility, most notably, fertilizers (Mårald et al. 2017). The application of mixed forest can also be compared to the application of clearcutting. Clearcutting has had a substantial support among Swedish foresters during the entire twentieth century but was not applied on large scales until the 1950s and 1960s (Lundmark 2020). In this context, clearcutting, alongside fertilizers and monocultures, but not mixed forests, seemed to fit perfect with the development of large-scale and “modern” forestry. Additionally, in difference to Hesselman’s ideas about monocultures, his ideas about clearcutting where quickly inserted into programmatic agendas by forestry authorities launching modern forestry (e.g., Ebeling 1955). Finally, contrasting with Germany, from where Hesselman gained his inspiration, interesting patterns also seem to appear. While both Swedish and German foresters saw mixed forests as a viable option, the latter were the only ones putting them into practice, at least at some scales during the 1920s and 1930s (Grewe and Hölzl 2018). Exploring causes and effects behind such differences in theory and practice would be a productive way forward.

## References

[CR1] Andersson G (1903). Skogssköfling och skogsodling i Cevennerna. Skogsvårdsföreningens tidskrift.

[CR2] Andersson G, Hesselman H (1907). Vegetation och flora i Hamra kronopark: Ett bidrag till kännedomen om den svenska urskogen och dess omvandling. Meddelanden från Statens skogsförsöksanstalt.

[CR3] Appelstrand, M. 2007. Miljömålet i skogsbruket: Styrning och frivillighet. PhD Thesis. Lund: Lund University.

[CR4] Balogh, A. 2021. The rise and fall of monoculture farming. *Horizon: The EU Research & Innovation Magazine*. https://ec.europa.eu/research-and-innovation/en/horizon-magazine/rise-and-fall-monoculture-farming. Accessed 9 Aug 2023.

[CR5] Bauhaus J, Puettmann KJ, Kühne C, Messier C, Puettmann KJ, Coates KD (2013). Close-to-nature forest management in Europe: Does it support complexity and adaptability of forest ecosystems?. Managing forests as complex adaptive systems: Building resilience to the challenge of global change.

[CR6] Björck H (2008). Folkhemsbyggare.

[CR7] Bruno K, Lundin P, Bruno K, Lundin P (2020). Inledning. Önskad och ifrågasatt: Lantbruksvetenskapernas akademisering i 1900-talets Sverige.

[CR8] Demeritt D (2001). Scientific forest conservation and the statistical picturing of nature’s limits in the Progressive-era United States. Environment and Planning D.

[CR9] Ebeling F (1955). Norrländska skogsvårdsfrågor: Kompendium i skogsskötsel för statens skogsskolor i Norrland.

[CR10] Eliasson P, Runefelt L (2008). Skogsdikning och skogsväxt under 1900-talet. Svensk mosskultur: Odling, torvanvändning och landskapets förändring.

[CR11] Eliasson P, Törnlund E, Oosthoek KJ, Hölzl R (2018). Swedish state forestry, 1917–2000. Managing northern Europe’s forests: Histories from the Aae of improvement to the age of ecology.

[CR12] Eriksson G (1978). Kartläggarna: Naturvetenskapens tillväxt och tillämpningar i det industriella genombrottets Sverige 1870–1914.

[CR13] Grewe, B-S. and Hölzl, R. 2018. Forestry in Germany, c. 1550–2000. In *Managing northern Europe’s forests: Histories from the Aae of improvement to the age of ecology*, eds. K. J. Oosthoek and R. Hölzl, 15–65. New York: Berghahn Books.

[CR14] Haraldsson, D. 1987. Skydda vår natur! Svenska naturskyddsföreningens framväxt och tidiga utveckling. PhD Thesis. Lund: Lund University Press.

[CR15] Hesselman, H. 1904. Zur Kenntnis des Pflanzenlebens schweischer Laubwiesen: Eine phsysiologisch-biologische und pflanzengeographische Studie. PhD. Thesis. Jena: Mitteilungen aus dem botanischen Institut der Universität zu Stockholm.

[CR16] Hesselman H (1905). Svenska lövängar. Skogsvårdsföreningens tidskrift.

[CR17] Hesselman H (1906). Om svenska skogar och skogssamhällen.

[CR18] Hesselman H (1911). Skabbholmen, en af Sveriges vackraste löfängar. Sveriges natur.

[CR19] Hesselman H (1917). Om det inflytande som våra skogsvårdsåtgärder kunna utöva på skogsmarkens alstringsförmåga.

[CR20] Hesselman H (1917). Om våra skogsföryngringsåtgärders inverkan på salpeterbildningen i marken och dess betydelse för barrskogens föryngring. Meddelande från Statens skogsförsökanstalt.

[CR21] Hesselman H (1917). Studier över salpeterbildningen i naturliga jordmåner och dess betydelse i växtekologiskt avseende. Meddelanden från Statens skogsförsöksanstalt.

[CR22] Hesselman, H. 1917d. Om det inflytande, som våra skogsvårdsåtgärder kunna utöva på skogsmarkens alstringsförmåga. *Skogen* 4: 1–12, 73–82, 165–185.

[CR23] Hesselman H (1918). Ljunghedslandskapet, ett försvinnande drag i svensk natur. Sveriges natur.

[CR24] Hesselman H (1919). Naturforskningen och de skogsbiologiska problemen. Skogsvårdsföreningens tidskrift.

[CR25] Hesselman H (1922). Moderna strömningar i Mellaneuropas skogsvård och deras betydelse för Sverige. Skogen.

[CR26] Hesselman H (1925). Studier över barrskogens humustäcke, dess egenskaper och beroende av skogsvården. Meddelanden från Statens skogsförsöksanstalt.

[CR27] Hesselman H (1937). Om humustäckets beroende av beståndets ålder och sammansättning i den nordiska granskogen av blåbärsrik *Vaccinium*-typ och dess inverkan på skogens föryngring och tillväxt. Meddelanden från Statens skogsförsöksanstalt.

[CR28] Hölzl R (2010). Historicizing sustainability: German scientific forestry in the eighteenth and nineteenth centuries. Science as Culture.

[CR29] Hughes D (2016). What is environmental history?.

[CR30] Imort M, Lekan T, Zeller T (2005). A sylvan people: Wilhelmine forestry and the forest as a symbol of Germandom. Germany’s nature: Cultural landscapes and environmental history.

[CR31] Jönsson, J. 2019. Den biologiska vändningen: Biologi och skogsvård, 1900–1940*.* PhD Thesis. Lund: Department of Arts and Cultural Sciences, Lund University.

[CR32] Jönsson J, Mårald E, Lundmark T (2021). The shifting society syndrome: Values, baselines, and Swedish forest conservation in the 1930s and 2010s. Conservation Science and Practice.

[CR33] Josefsson T, Östlund L, Antonsson H, Jansson U (2011). Increased production and depletion: The impact of forestry on northern Sweden’s forest landscape. Agriculture and forestry in Sweden since 1900: Geographical and historical studies.

[CR34] Langston N (1995). Forest dreams, forest nightmares: The paradox of old growth in the Inland Northwest.

[CR35] Larsson Heidenblad D (2021). The environmental turn in postwar Sweden: A new history of knowledge.

[CR36] Lisberg Jensen E, Antonsson H, Jansson U (2011). Modern clear-felling: From success to story to negotiated solution. Agriculture and forestry in Sweden since 1900: Geographical and historical studies.

[CR37] Loo T, Stanley M (2011). An environmental history of progress: Damming the peace and Colombia rivers. Canadian Historical Review.

[CR38] Lowood H, Frängsmyr T, Heilbron JL, Rider RE (1990). The calculating forester: Quantification, cameral science, and the emergence of scientific forestry management in Germany. The quantifying spirit in the Eighteenth Century.

[CR39] Lundgren, L.J. 2009. *Staten och naturen: Naturskyddspolitik i Sverige 1869–1935: Del I: 1869–1919*. Brottby: Kassandra,

[CR40] Lundmark H (2020). Clear-cutting: The most discussed logging method in Swedish forest history.

[CR41] Mårald E, Langston N, Sténs A, Moen J (2016). Changing ideas in forestry: A comparison of concepts in Swedish and American forestry journals during the early twentieth and twenty-first centuries. Ambio.

[CR42] Mårald E, Nordlund C (2020). Modern nature for a modern nation: An intellectual history of environmental dissonances in the Swedish welfare state. Environment and History.

[CR43] Mårald E, Sandström C, Nordin A (2017). Forest governance and management across time: Developing a new social contract.

[CR44] Mårald E, Westholm E (2016). Changing approaches to the future in Swedish forestry, 1850–2010. Nature and Culture.

[CR45] Metoder för naturlig föryngring. 1924. *Skogen* 10: 173–215.

[CR46] Nature-based solutions: A tool for climate adaptation and other societal challenges. 2022. Swedish Environmental Protection Agency, Report 7074, Stockholm, Sweden.

[CR47] Östlund L, Norstedt G (2022). Preservation of the cultural legacy of the indigenous Sami in northern forest reserves: Present shortcomings and future possibilities. Forest Ecology and Management.

[CR48] Puettmann KJ, Coates D, Messier C (2008). A critique of silviculture: Managing for complexity.

[CR49] Radkau J (2012). Wood: A history.

[CR50] Scott JC (1998). Seeing like a state: How certain schemes to improve the human condition have failed.

[CR51] Skinner Q (2002). Visions of politics: Volume 1: Regarding method.

[CR52] Söderqvist T (1986). The ecologists: From merry naturalists to saviours of the nation.

[CR53] Sörlin S (2011). The contemporaneity of environmental history: Negotiating scholarship, useful history and the new human condition. Journal of Contemporary History.

[CR54] Sundin B (2006). Nature as heritage: The Swedish case. International Journal of Heritage Studies.

[CR55] Uekötter F (2011). The magic of one: Reflections on the pathologies of monoculture. RCC Perspectives.

[CR56] Worster D (1994). Nature’s economy: A history of ecological ideas.

